# Proton-Initiated Reversible Chalcogen-Vertex Extrusion in Macropolyhedral Chalcogenaboranes

**DOI:** 10.1021/acs.inorgchem.5c03815

**Published:** 2026-02-18

**Authors:** Jonathan Bould, Miroslava Litecká, William Clegg, Marcel Ehn, John D. Kennedy, Michael G. S. Londesborough

**Affiliations:** † Institute of Inorganic Chemistry of the Czech Academy of Sciences, 250 68 Husinec-Řež č.p. 1001, Czech Republic; ‡ School of Natural and Environmental Sciences, Newcastle University, Newcastle upon Tyne NE1 7RU, U.K.; § School of Chemistry, University of Leeds, Leeds LS2 9JT, U.K.

## Abstract

Protonation of the macropolyhedral chalcogenaborane anions [E_2_B_17_H_18_]^−^ (E = S, **1a** ^**–**^ Se, **1b** ^**–**^) induces a radical cluster rearrangement, yielding the new metastable isoelectronic neutral species E_2_B_17_H_19_ (**2a** and **2b**). This transformation converts the original structure comprising two identical “*arachno*” 10-vertex subclusters into a new architecture with two distinct “*nido*” 10-vertex subclusters. Notably, one of the chalcogen atoms, which originally was a triconnected cluster vertex in anions **1** ^**–**^, is extruded, forming a bridging μ_2_-{EH} unit. Remarkably, deprotonation of compounds **2** facilitates a boronotropic dehydrogenation, giving reintegration of the extruded {EH} unit and regeneration of the original [E_2_B_17_H_18_]^−^ anions **1** ^**–**^. Compound **2a** is characterized by a single-crystal X-ray diffraction (SCXRD) study, and by agreement between experimental and density functional theory (DFT)-calculated multielement NMR spectra. Also, for **2b**, the measured and calculated δ­(^77^Se) values for the two widely separated selenium resonances add further support. Metastable compounds **2** ultimately eliminate dihydrogen to yield the known neutral E_2_B_17_H_17_ compounds **3**. For **2b**, a competing pathway leads to loss of selenium, to give known SeB_17_H_19_ **4**.

## Graphical abstract

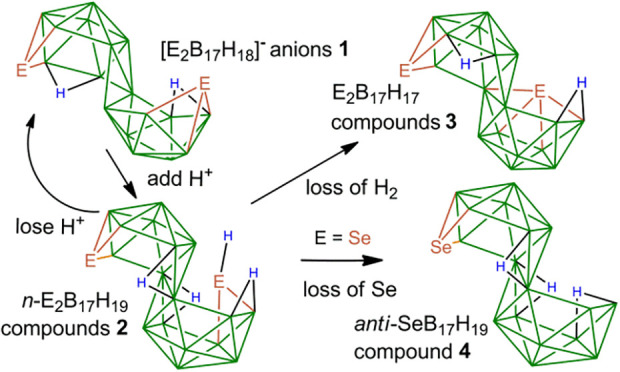

## Introduction

Polyhedral boron hydride chemistry occupies a distinctive position within inorganic chemistry, offering rare insights into electron-delocalized cluster architectures, unusual bonding motifs, and a capacity for structural adaptability that rivals the diversity seen in organic frameworks.^1^ Among these compounds, macropolyhedral chalcogenaboranes have emerged as particularly intriguing systems, not least because the incorporation of chalcogens perturbs the delicate balance between electron count, three-dimensional (3D) architecture, reactivity, and photophysical properties of the borane cluster core.^2^ The anions [E_2_B_17_H_18_]^−^ (E = S, Se) exemplify this complexity.^2b,3^ They consist of two equivalent *arachno* 10-vertex subclusters linked by two common boron atoms (see Scheme 1), situating them at the intersection of classical Wade–Mingos electron-counting concepts^2^ and the more flexible behavior exhibited by large, maropolyhedral metallaboranes, categorized by Jemmis et al.^4^

**Scheme 1:**
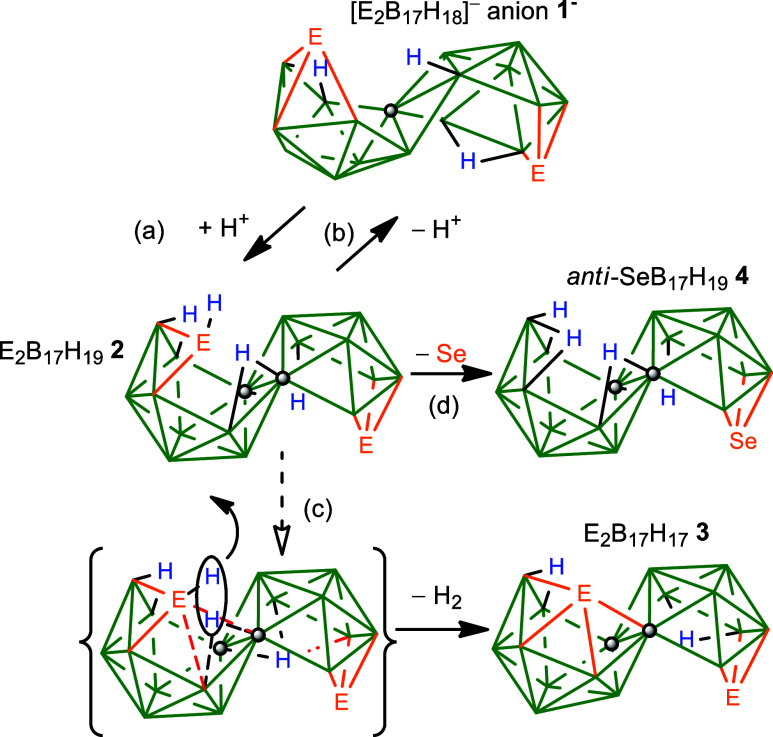
Overall Reaction Pathways^a^

a (a) Protonation of the [E_2_B_17_H_18_]^−^ anions **1** ^**–**^ with extrusion of an E vertex as a B–B^μ_2_^-{EH} bridging unit to quantitatively give neutral E_2_B_17_H_19_ **2**. (b) Deprotonation of E-H in compounds **2** to regenerate anions **1** ^**–**^. (c) Dihydrogen loss from **2** and reincorporation of the E atom into the open face of its 10-vertex *nido*-{B_10_} subcluster generates the *nido* 11-vertex {EB_10_} subcluster in compounds E_2_B_17_H_17_ **3**, E = S: 100%.^2b,3b^ (d) Selenium-atom loss from the μ_2_-{SeH} unit, when E = Se, but with retention of hydrogen, generates *anti*-SeB_17_H_19_, **4**, 10%.^2b^ When E = S, formation of *anti*-SB_17_H_19_ is not observed. Grey circles represent *commo* boron atoms with no *exo*-terminal hydrogen atoms. All other boron vertices hold a terminal hydrogen atom. Red hashed lines represent new connectivities formed during the transitions of species **2** to **3**.

Comparison with carborane chemistry provides further context. Carboranes, particularly *closo*-C_2_B_10_H_12_ and its derivatives, are renowned for their structural resilience and for the rich, position-specific substitution chemistry made possible by their rigid icosahedral scaffolds.^5^ Large-scale topological rearrangements are rare in carboranes, reflecting the exceptional stability of the polyhedral cage. In contrast, the heteroboranes considered here display a striking structural plasticity: as we shall show, a single proton triggers a profound rearrangement that reshapes the macropolyhedral topology. This divergence illustrates how chalcogen incorporation unlocks new modes of reactivity inaccessible to purely boron–carbon clusters.

An understanding of the structure and behavior of metastable intermediates is crucial for elucidating mechanistic pathways in such complex reorganizations. Although transient species are widely recognized as key actors in organic and organometallic chemistry, their roles in boron macropolyhedral cluster transformations remain comparatively underexplored. The acid-induced structural dynamism of the [E_2_B_17_H_18_]^−^ anions, where E refers to sulfur (**a**) or selenium (**b**) as appropriate, provides a compelling case study and is the subject of our work here. The [S_2_B_17_H_18_]^−^ anion (**1a** ^**–**^), first described in 1994,^3a^ was reported then to seemingly undergo immediate conversion to S_2_B_17_H_17_ (**3a**) upon protonation, accompanied by a notable ∼33° rotation in the relative orientation of the two *arachno* subclusters. We later showed that the selenium analogue [Se_2_B_17_H_18_]^−^ (**1b** ^**–**^) displays similar reactivity.^2b^ In both cases, however, our contemporary work, which benefits from the possibilities afforded by modern NMR spectroscopic studies of the reaction, revealed the fleeting formation of metastable 19-vertex intermediates, now identified as E_2_B_17_H_19_ (**2a** E = S; **2b** E = Se), bridging the transformation of **1** into **3**. Despite repeated observation of these species in our previous NMR studies, the intrinsic instability of these species has, until now, prevented their definitive structural characterization.

## Results and Discussion

Acidification of dichloromethane solutions of the [E_2_B_17_H_18_]^−^ anions **1** with concentrated H_2_SO_4_ yields neutral E_2_B_17_H_19_ compounds **2**, which, over several hours, undergo spontaneous loss of dihydrogen to give neutral E_2_B_17_H_17_ **3** and, in the case of selenium, a small amount of EB_17_H_19_ **4** (Scheme 1). From a single-crystal X-ray diffraction study (SCXRD) the molecular structure of **2a** (Figure 1) is seen to exhibit a {μ_2_-SH}-SB_17_H_18_ unit. The configuration is analogous to that of the long-known *anti*-B_18_H_22_, but with an {SH} unit bridging an open-face B–B link in a position occupied by a bridging hydrogen atom in *anti-*B_18_H_22_ (Schematic structures **I** and **II** in Figure 1). Bridging thiol groups are rare in polyhedral borane chemistry, with only S_2_B_16_H_18_ ^6^ and mercaptoboranes such as μ_2_-HS­(B_2_H_5_)^7^ as known exemplars. In a further analogy with *anti*-B_18_H_22_, the structure of **2a** can be viewed as two “*nido*” 10-vertex subclusters that are fused with two boron atoms held in common.

**Figure 1:**
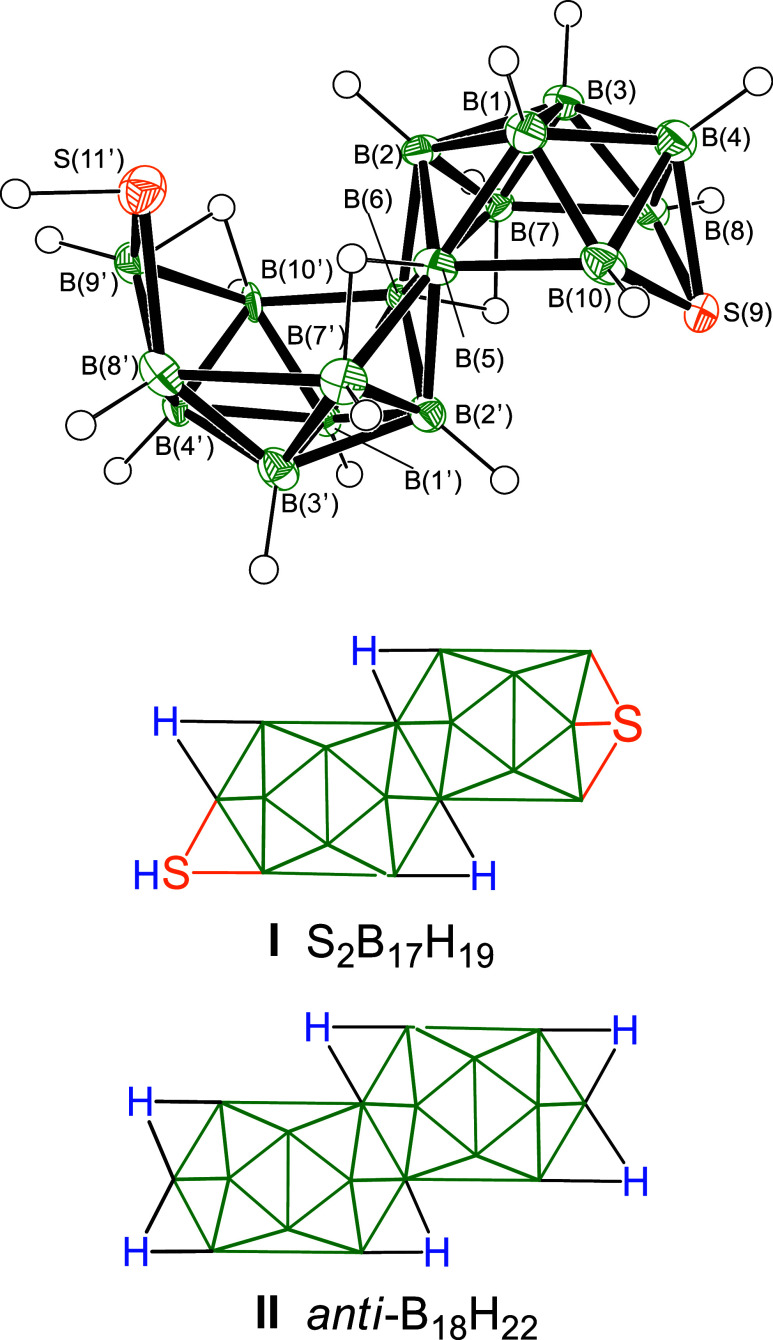
Above: Crystallographically determined structure of the 19-vertex dithiaborane S_2_B_17_H_19_ **2a** with 50% probability ellipsoids for non-hydrogen atoms. Below: schematic diagram comparing projections of the basic cluster architectures of S_2_B_17_H_19_ **2a** and *anti*-B_18_H_22_. The selenium analogue **2b** is isostructural.

The results from the SCXRD study are substantiated by the close agreement between the experimental ^11^B NMR data for **2a** and the values predicted via density functional theory (DFT)/GIAO calculations for the molecular structure and boron nuclear shieldings (Tables S1 and S2). Comparison of the calculated and measured ^11^B NMR spectra for **2a** is in Figures S1 and S2. Further substantiation derives from comparing the ^11^B spectra of the now substantiated selenium analogue **2b** with SeB_17_H_19_ **4** (Figure 2), which show that the effective replacement of a bridging hydrogen atom in **4** by {SeH} results in the largest changes in resonance positions for those vertices associated with the bridging {SeH} unit. Further, the measured ^77^Se NMR spectrum of **2b** (Figure S4) exhibits two resonances at +400 and −390 ppm, which correspond to Se(9) and Se(11′) respectively in Figure 1. To support the characterization of **2b**, we employed published correlations between measured ^77^Se NMR chemical shifts and DFT/mPW1PW91-calculated selenium nuclear shieldings for a range of selenaborane species.^8^ These correlations have proven valuable in substantiating measured ^77^Se chemical shifts where direct structural data are unavailable for new species, and thence allowing structural conclusions. For **2b**, the SCXRD data were suboptimal because of twinning and disorder and their refinement involved the support of the DFT structural model, particularly with regard to the positions of bridging and selenol hydrogen atoms. The calculated ^77^Se chemical shifts for the proposed structure of the selenium analogue **2b** (Figure 3) are thence in agreement with the experimental values, thereby providing strong extra support for the structure. The assignments of the selenium and boron resonances might, in principle, be further strengthened by the observation of ^77^Se satellites on the boron resonances and correlating these with their assignments from DFT calculation. However, the line widths for ^11^B and ^77^Se are of the order of 80 and 150–300 Hz respectively, and any selenium satellites are very weak, 3.5% either side of the resonances, and are too broad to observe.

**Figure 2:**
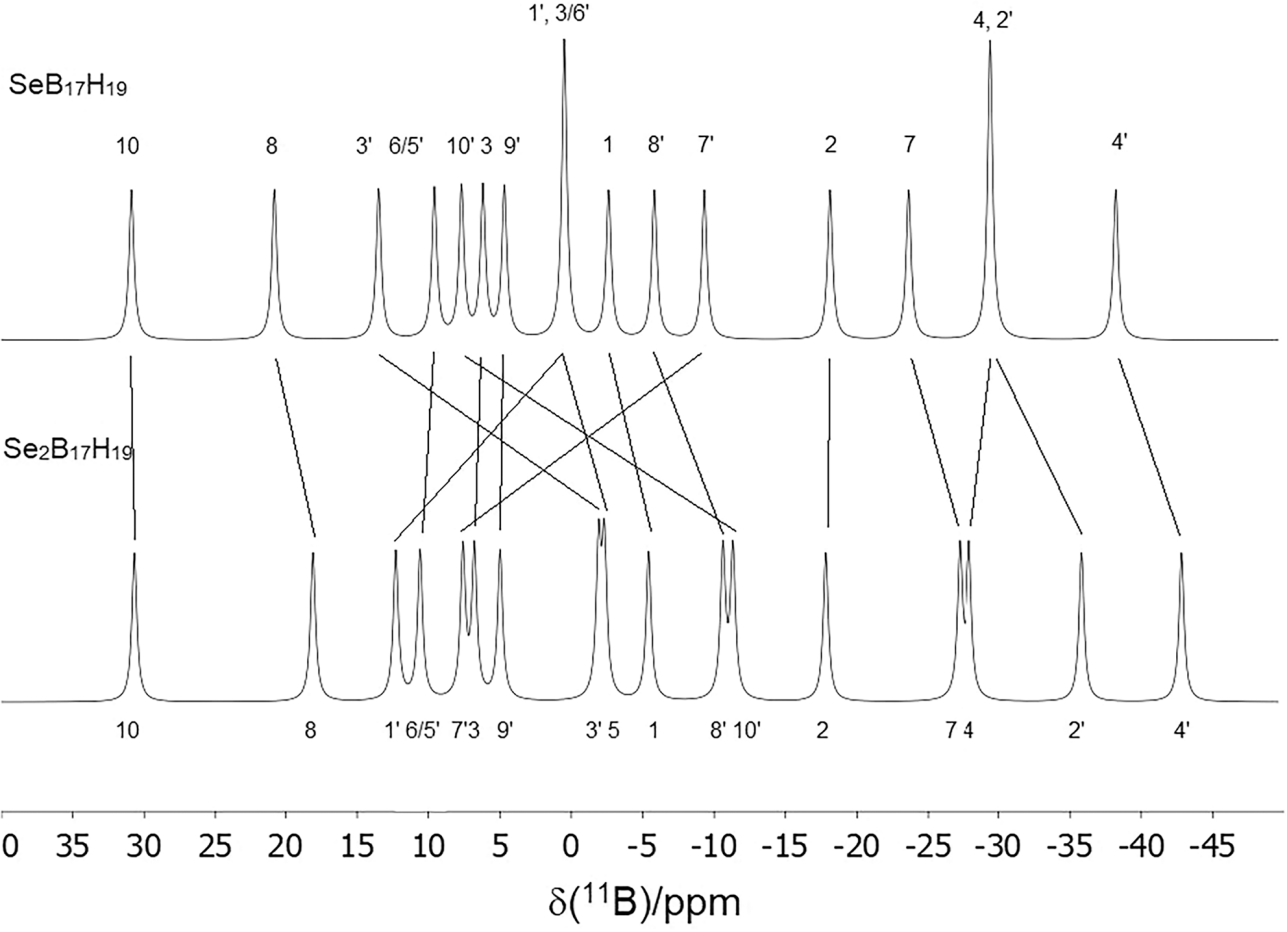
An illustration (with arbitrary peak widths) of the relative positions of the measured ^11^B-{^1^H} resonances in SeB_17_H_19_ **4** ^2b^ and Se_2_B_17_H_19_ **2b** emphasizing the changes induced by the bridging {μ-SeH} moiety in **2b**. It may be noted that there is only a small change in the chemical shift of the B(4′) position, which is *trans* to the {μ_2_-SeH} moiety, in going from SeB_17_H_19_ to Se_2_B_17_H_19_. The substitution of the bridging hydrogen atom in SeB_17_H_19_ **4** with the {μ-SeH} moiety might be expected to produce a more significant effect. The sulfur analogue of SeB_17_H_19_ **4** does not yet exist and so a direct comparison is not possible. However, the calculated relative chemical shifts for the 4′ positions in SB_17_H_19_ and S_2_B_17_H_19_ pair also show the same 5 ppm upfield shift. This suggests that the B(4′) chemical shift is relatively insensitive to the identity of E.

**Figure 3:**
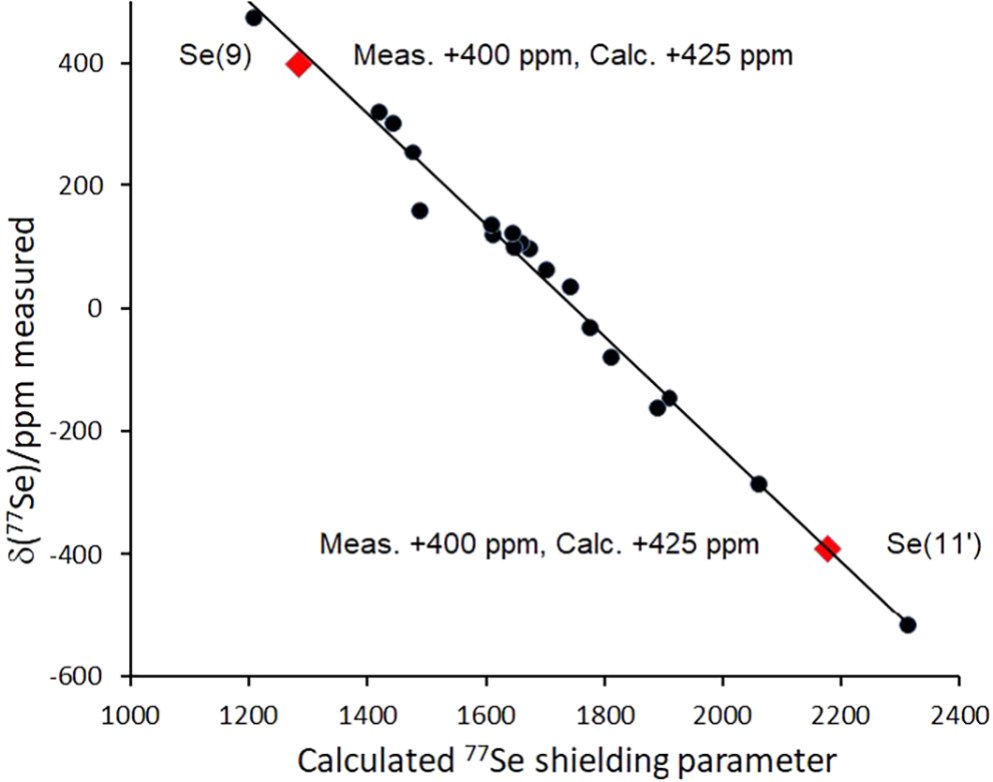
Correlation of the experimentally determined ^77^Se NMR chemical shifts and the mPW1PW91–calculated selenium magnetic nuclear shieldings for a range of macropolyhedral and single-cluster selenaboranes. The 19 selenium centers (black circles) range over 1000 ppm.^2b,8^ The points for the two selenium vertices in now-substantiated Se_2_B_17_H_19_ **2b** are shown as orange diamonds. The trend line is calculated from the published data^2b,8^ and does not include compound **2b**. The linear regression analysis gives δ_calc_ = *A* × δ_exp_ + *B* 0.906*x* + 21.4, *R* ^2^ = 0.989. The measured highly shielded Se–H chemical shift at −390 ppm compares to that in SeH_2_ of −345 ppm,^9^ which might support its characterization as a “*pseudo* bridging-hydrogen”.

The mechanistic implications of the four transformations–*viz*. the processes (a) to (d) in Scheme 1–that ultimately lead to the effective 33° cluster subcluster reorientations, as previously noted,^3b^ in the overall conversions of species of type **1** ^**–**^ to type **3**, may now be rationalized. In this context, the structural elucidation of the intermediate species **2** allows the following proposals:

First, regarding the transition of anions **1** ^**–**^ to compounds **2**, process (a).

1. That **2a** features an {H–S} unit suggests that the most probable site for the initial protonation of compounds **1** ^**–**^ is the E atom.
2. For the sulfur species **1a** ^**–**^, its initially protonated entity, neutral **1a**′, is calculated to be 19.5 kcal/mol higher in energy than the resulting metastable isomer **2a**′, providing a considerable driving force for the transformation process (Scheme 2).

**Scheme 2:**
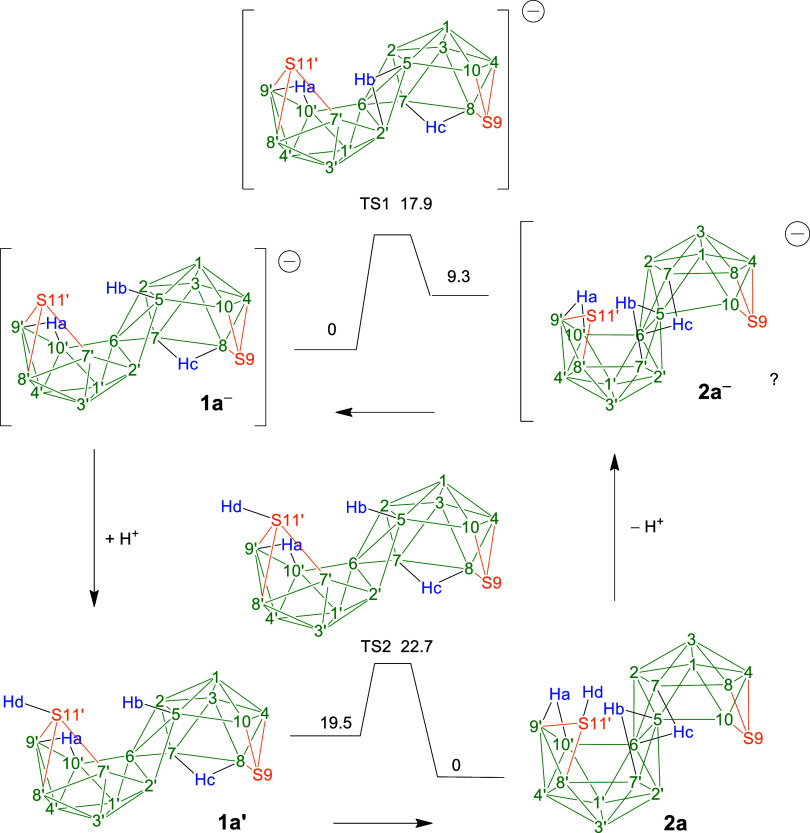
Schematic Structures to Show the Proposed Relative Positions of the Vertices and Bridging Hydrogen Atoms, with Energies as Calculated for the Sulfur System as Exemplars^a^

a The behavior of the selenium system will be similar. Thus, deprotonation of S_2_B_17_H_19_ **2a** at the S11′ position (bottom right) affords the [S_2_B_17_H_18_]^−^ anion **2a** ^–^. This rearranges *via* transition state 1 (TS1) to give **its** isomer **1a** ^**–**^ (top left) which is 9.3 kcal/mol lower in energy than **2a** ^–^. Protonation of **1a** ^**–**^ at S11′ (top left) then produces, via TS1, neutral **1a′** which is 19.5 kcal/mol higher in energy than its structural isomer S_2_B_17_H_19_ **2a**, to which it reverts, via TS2. The numbering in the clusters corresponds to that used in Figure 1. See Supporting Information TS1 and TS2 for transition state animations.

Thus, protonation of one of the chalcogen atoms in **1** ^–^ to give compounds **1**′ triggers an extrusion of this vertex from its original three-connected, cluster-integrated position to a more exposed {μ-EH}-bridging unit in compounds **2**. This displacement of one E atom as {EH} reduces the formal cluster bonding electron count by three, assuming that the three-connected E vertices in compounds **1** ^**–**^ act as 4-electron donors, while the bridging {EH} units behave as one-electron, *pseudo*-hydrogen bridges: the nature of bridging heteroatoms in boranes as bridging or as vertices has been discussed in detail elsewhere.^10^ The rearrangement involves cleavage of the S(11)–B(7) connectivity **1a** ^**–**^ (in Scheme 2) and formation of a new B(7)–B(5) linkage in **2a**. The substantial reorganization of the framework prompts the *exo*-hydrogen atom BH(5) (Hb in **1a** ^**–**^) to shift position, now to function as a bridging μ-H­(7′,5) hydrogen atom across the newly established B–B linkage. In doing so, the delocalized negative charges present in anions **1** ^**–**^ become localized as μ-H­(7,5) bridges, increasing the total number of bridging hydrogen atoms from two in anions **1** ^**–**^ to three in compounds **2**. Accordingly, process (a) represents a formal oxidative cluster transformation, effecting a geometrical shift from *arachno* to *nido* in both subclusters of compounds **2**. It also results in the migration of the μ-H­(7,8) bridge (Hc in anions **1** ^**–**^) to a μ-H­(6,7) position in both compounds **2** (Scheme 2).

The extrusion of the heteroatom cluster vertex to a bridging position is unusual enough to warrant a brief discussion. Thiolated derivatives of the single-cluster species B_10_H_14_ and of the isomers of macropolyhedral B_18_H_22_ are known.^11^ These compounds feature thiol groups that have replaced, by Friedel–Crafts–type electrophilic substitution, *exo*-polyhedral hydrogen atoms on the periphery of an unchanged boron skeleton. This is in contrast to the current case where the thiol group arises from an internal migration and partial ejection from the cluster. A bridging sulfur has been observed^12^ in the 18-vertex species *iso*-S_2_B_16_H_16_ in which the sulfur may be regarded as replacing both the bridging hydrogen atom in one subcluster and the *exo*-terminal hydrogen atom in the adjacent subcluster (Scheme 3). Here the bonding could be described as comprising one 2-electron 3-center bond and one 2-electron 2-center bond. Architecturally, the sulfur atom in *iso*-S_2_B_16_H_16_ could be regarded as resembling a thioether.^13^ However, the thiol motif in **2** is not fully comparable to either a thioether or a thiiriane as the latter is an S–C–C 3-membered ring with the sulfur bonded to the carbon atoms via two strong 2-center, 2-electron bonds. Also, in thiiranes, the S bridges a C–C 2-electron σ-bonded linkage whereas in **2a** the B8′-B9′ connectivity at 2.058(10) Å (1.92 Å in *iso*-S_2_B_16_H_16_) is looser than a direct B–B sigma linkage which would be expected to be *ca*. 1.6 Å, and the bridged two boron atoms are part of a diffuse multicenter bonding matrix.

**Scheme 3:**
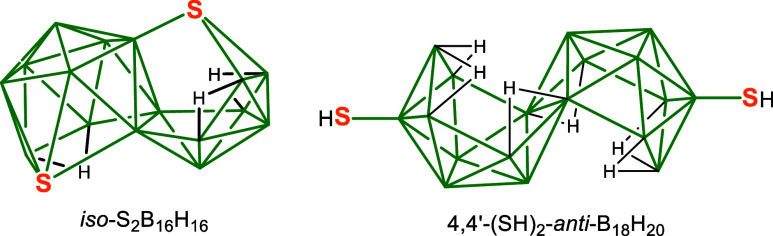
Schematic Structures of *iso*-S_2_B_16_H_16_ and 4,4-(HS)_2_-*anti*-B_18_H_20_

Second, regarding the reversion of **2a** into **1a** ^**–**^, DFT calculations show, for the sulfur exemplar:

1. Deprotonation at the {H–S} position in **2a** produces an anion, [S_2_B_17_H_18_]^**–**^ (**2a** ^**–**^), which is 14.6 kcal/mol lower in energy than for the deprotonation of any of the cluster bridging hydrogen atoms in **2a** (see below), and thus is the most likely candidate for deprotonation in compounds **2**.
2. This [S_2_B_17_H_18_]^**–**^ anion **2a** ^**–**^, from deprotonation of μ-{H–S}, is 9.3 kcal/mol higher in energy than isomeric [S_2_B_17_H_18_]^**–**^ anion **1a** ^**–**^ into which it rearranges via transition state 1 (TS1).

The hydrogen atoms bridging to the *commo* boron atoms in *anti-*B_18_H_22_ are known to be highly acidic,^14^ and compounds **2**, as analogues of *anti-*B_18_H_22_, likewise each hold three acidic bridging hydrogen atoms. Among the two possible bridging hydrogen atoms, DFT calculations reveal that deprotonation of H­(6,7′) on **2** (Hc on **2a** in Scheme 2) yields the lowest-energy structure. However, rather than deprotonation at a bridging site, deprotonation at the {SH} site (Hd in **2a**) leads to formation of the [S_2_B_17_H_18_]^**–**^ anion **2a** ^**–**^, with a bare bridging sulfur atom, which, as summarized above, is 14.6 kcal/mol lower in energy than the H­(6,7′)-deprotonated anion (Hc). Thence, the anion **1a** ^**–**^ is 9.3 kcal/mol lower in energy than the isomeric **2a** ^**–**^ from which it rearranges. This rearrangement involves the breaking of the B(7′)-B(5) connectivity and reformation of the S­(11′**)–**B**(**7′) linkage. The increased integration of the chalcogen atom into the cluster bonding framework and the resultant injection of electron density reflect in the open-face hydrogen-atom reorganization.

Third, regarding the transformation of compounds **2** into compounds **3**, process (c) in Scheme 1:

The bridging {EH} unit can subsequently reinsert into a more intimate cluster environment upon loss of dihydrogen, ultimately forming the 19-vertex E_2_B_17_H_17_ **3** (Scheme 1). This reinsertion involves formation of new E(11′)–B(7′) and E(11′)–B(5) connectivities. Intuitively, the eliminated H_2_ molecule derives from the μ-H­(7′,5) proton and the {EH} hydride. Indeed, protonation of anion **1a** ^**–**^ with D_2_SO_4_ ultimately produces S_2_B_17_H_17_ **3a** in which all *exo*-terminal and bridging atoms are hydrogen, i.e., the deuterium on sulfur is lost as HD. The required structural transition would involve the *exo*-{E–H} thiol/selenol unit (shown in Figure 1) adopting an inwardly pointing *endo*-{E–H} polyhedral vector, thereby enabling a configuration that facilitates dihydrogen loss to afford compounds **3** as illustrated with the encircled hydrogen atoms in Scheme 1(c). DFT calculations show that the prospective species with *endo* **-**polyhedral orientation for S_2_B_17_H_19_ **2a** are only 3 kcal/mol higher in energy than the *exo*-terminal configuration; the low *end*o-to-*exo* barrier of 11.2 kcal/mol for S_2_B_17_H_19_ indicates a feasible transition for this postulated first step. It may be noted here, with regard to the refinement of the SCXRD data and the derived positions of the hydrogen atoms, that calculated selenium magnetic nuclear shielding data for the hypothetical **2b** species featuring an *endo*-polyhedral {Se–H} hydrogen yields a δ­(^77^Se) value of −500 ppm, approximately 100 ppm more shielded than the experimental and calculated values for **2b**(*exo*); this therefore eliminates the *endo* possibility in the solid state structural model. This emphasizes the value of accurate chemical shielding predictions in selenaborane systems for confirming structural assignments.^8^

Finally, and uniquely in the case of selenium, a competing process emerges –process (d) –in which the chalcogen atom can be eliminated altogether, with retention of its associated {SeH} hydrogen atom, leading to the 18-vertex SeB_17_H_19_ **4**, as we have previously reported.^2b^ This competing pathway does not occur to any observable extent in the corresponding sulfur system.

## Conclusion

The isolation and structural characterization of the metastable 19-vertex S_2_B_17_H_19_ **2a** provides the identity of the intermediate in the acid-mediated transformation of anions [E_2_B_17_H_18_]^−^ **1** ^**–**^ to the known neutral chalcogenaboranes E_2_B_17_H_17_ **3**,^2b,3b^ the remarkable luminescent properties of which have only recently been described.^2b^ This rearrangement involves an unusual, and unusually reversible, extrusion of a chalcogen vertex, accompanied by significant topological reorganizations of the boron framework from “*arachno*” to “*nido*” character in both subclusters. The ability of **2a** and **2b** to undergo this reversible structural reorganization—including reintegration of the extruded {EH} unit via dehydrogenation—underscores the dynamic and versatile nature of chalcogenaborane clusters. The reversibility of this rearrangement positions this chemistry within a broader trend of *boronotropic* processes, wherein proton transfer events mediate skeletal reintegration and molecular “self-repair”. This behavior contrasts with many classical borane rearrangements, such as the protonation of the polyhedral anion [*closo*-B_10_H_10_]^2–^ under superacidic conditions,^15^ which are typically irreversible once the cluster has relaxed into a lower-energy polyhedron.

To provide further context, there are a number of base-induced extrusion and reinsertion of vertices in metallaborane clusters that have previously been observed on the addition or removal of certain Lewis bases (L = PMe_2_Ph or PPh_3_). For example, [(PPh_3_)_2_CO-*nido*-B_5_H_9_] affords [(PPh_3_)­CO-*exo-arachno*-B_4_H_8_-(BH_2_•L)] on addition of phosphine.^16^ Similarly, Lewis base induced vertex isomerizations have been reported in 10-vertex *nido*-metalladecaboranyl species such as 8-[(C_2_H_5_)_3_N­(CH_2_)_4_O]-6-(CO)_3_-6-(CO)_3_-6-MnB_9_H_12_ and [6,6,6-(PPh_3_)_2_CO-*nido*-6-B_9_H_13_] which were rationalized by intramolecular vertex “shifts”^17^ or “swings”^18^ around the cluster and, in the case of [9-(C_5_Me_5_)-*nido*-6,9-NRhB_8_H_11_], a thermally induced rearrangement via a proposed transition state involving the extrusion of one cluster vertex. More pertinent to the work here, cluster isomerizations induced by protonation, and reversed by deprotonation have been observed in the dicarbadecaboranyl species X-*nido*-5,6-C_2_B_8_H_11_ (X = Cl, Br, I), with the proposed mechanism supported by DFT calculations.^19^ Here the authors proposed intramolecular rearrangements to rationalize the products and they excluded the vertex swing or extrusion mechanisms.

Thus, the E_2_B_17_H_19_ **2** species are examples that combine aspects of both features, the extrusion of a vertex induced by protonation and deprotonation. Taken together, these results demonstrate that chalcogen substitution enables a previously inaccessible regime of reversible cluster editing, one that challenges traditional assumptions about borane rigidity. They demonstrate how the addition or removal of a single proton can propagate structural change across an entire macropolyhedral framework, offering new mechanistic insight into the dynamic behavior of electron-deficient clusters and suggesting future opportunities in the design of responsive molecular architectures. The structural and behavioral versatility compared to binary borane macropolyhedra is caused by the valence flexibility of sulfur and selenium as compared to the first-row element boron.

## Experimental Section

The preparation of S_2_B_17_H_19_ and Se_2_B_17_H_19_ essentially followed the published method for the preparation of E_2_B_17_H_17_ **3** and SeB_17_H_19_ **4**.^2b^ In an NMR tube a concentrated dichloromethane solution of the *N*,*N*′-dimethyl-naphthalenediamine (tmnd, Proton Sponge) salt of the [S_2_B_17_H_18_]^−^ anion (60 mg, 0.13 mmol in 0.4 mL CH_2_Cl_2_) was immersed in a cold bath (−20 to −30 °C) and concentrated H_2_SO_4_ (*ca*. 0.2 mL of 95%, VWR Chemicals BDH) was quickly added and the mixture then agitated. The boron spectrum was measured at −20 °C and the sample ejected from the spectrometer and warmed slightly in the hand and then reinserted. This was repeated until all the starting material had reacted to give the S_2_B_17_H_19_ metastable intermediate **2a** and before significant amounts of S_2_B_17_H_17_ **3a** had formed. The initial formation of **2a** is quantitative by NMR but the subsequent rate of **3a** formation increases at room temperature and the isolation of large amounts of pure **2a** is not feasible. Solutions of [tmnd]­[Se_2_B_17_H_18_] **1b** were treated similarly but this system is much more difficult to handle and to obtain reasonably impurity-free product **2b**. Many attempts were required for both **2a** and **2b** and no reliable isolated yields could be established. Single crystals for X-ray diffraction analysis were obtained by holding a highly concentrated dichloromethane solution of S_2_B_17_H_19_ **2a** in an NMR tube in a freezer at −25 °C for *ca* 2 weeks. A mass of yellow crystalline and semicrystalline material formed. The compound is very soluble in dichloromethane and so, while still cold, the NMR tube was broken open to drain away the solvent as rapidly as possible and before it could redissolve. A suitable crystal was discovered in the isolated mass of material.

### Notes on Acid Used

Sulfuric acid was found to be the most useful acid in practice. This formed a 2-phase mixture, which allowed the very simple decanting of the dichloromethane layer. Indeed, when cold, the acid is immobile and it allows to pour off the dichloromethane while the acid remains in the NMR tube. CF_3_COOH does protonate the anion but, as a liquid, it requires the removal of the solvent and the acid together by evaporation. This leads to deprotonation of compounds **2** by the Proton Sponge and reformation of **1**.

### NMR Spectroscopy

NMR spectra were recorded on a JEOL ECZ 600 R (14.1 T) spectrometer, using ^77^Se, ^11^B, ^11^B­{^1^H}, ^1^H, ^1^H­{^11^B­(broadband)}, ^1^H­{^11^B­(selective)}, and HMQC (Heteronuclear Multiple-Quantum Correlation) techniques. NMR spectra of all compounds were measured in CDCl_3_ solution. ^11^B chemical shifts are given relative to BF_3_·OEt_2_, δ^11^B = 0.0 ppm for Ξ­(^11^B) = 32,083,971 Hz. ^77^Se chemical shifts are reported relative to SeMe_2_, δ­(^77^Se) = 0.0 ppm for Ξ (^77^Se) = 19,071,523 Hz.

^77^Se NMR spectra were measured using a standard single-pulse sequence (available from the spectrometer library) with a 90° pulse length and relaxation delay of 0.1–0.2 s. The line widths of the ^77^Se resonances were in the range 200–300 Hz and the spectra were recorded with an FID resolution of 3–4 Hz.

### Computational Details

Calculations were performed using the Gaussian16 package.^20^ The DFT/B3LYP methodology was employed for the calculation of boron and hydrogen nuclear shieldings with the 6-31+G­(d,p) basis sets for B and H and the Binning and Curtiss 962+d polarization basis set for Se taken from BasisSetExchange.^21^ The selenium nuclear shielding calculations were carried using the same basis sets and the mPW1PW91 methodology. The Polarizable Continuum Model was implemented with CH_2_Cl_2_ solvation. Frequency calculations at the appropriate level confirmed the theoretical geometries as energy minima. Transition states TS1 and TS2 were calculated using the Synchronous Transit-guided Quasi-Newton method,^22^ QST3, and the B3LYP/6-31+G­(d,p) method and basis sets and confirmed by Intrinsic Reaction Coordinate (IRC) calculations. See Supporting Information for TS1 and TS2 animations.

### X-ray Crystallography

X-ray diffraction analysis was performed with an XtaLAB Synergy, Dualflex, HyPix diffractometer. The crystal was kept at 100 K during data collection. CrysAlisPro software was used for data collection and cell refinement, data reduction and absorption correction.^23^ Data were corrected for absorption effects using a semiempirical absorption correction (spherical harmonics), implemented in the SCALE3 ABSPACK scaling algorithm; neither a numerical absorption correction based on Gaussian integration over a multifaceted crystal model^24^ nor an analytical absorption correction made any significant difference to the data.^24,25^ The structure was solved with the ShelXT structure solution program^26^ using an iterative dual-space method and refined with the ShelXL^27^ refinement package using least-squares minimization, native and implemented in Olex2.^28^ Anisotropic displacement parameters were refined for all non-H atoms. The hydrogen atoms were calculated to idealized positions based on the DFT-calculated structure.

Crystallographic data for structural analysis have been deposited with the Cambridge Crystallographic Data Centre, CCDC no. 2452393. Copies of this information may be obtained free of charge from The Director, CCDC, 12 Union Road, Cambridge CB2 1EY, U.K. (fax: + 44–1223–336033; Email: deposit@ccdc.cam.ac.uk or http://www.ccdc.cam.ac.uk).

Crystal Data for S_2_B_17_H1_19_ **8** (M = 267.04 g/mol): monoclinic, space group *P*2_1_/*n* (no. 14), *a* = 21.9921(12) Å, *b* = 6.7157(2) Å, *c* = 22.1404(13) Å, β = 117.789(7)°, *V* = 2892.8(3) Å^3^, *Z* = 8, *T* = 100 K, μ­(Cu–Kα) = 2.947 mm^–1^, *D*_calc_ = 1.226 g/cm^3^, 8398 reflections measured (7.756° ≤ 2θ ≤ 154.206°, 5811 unique, *R*_int_ = 0.1681, *R*_sigma_ = 0.0203) which were used unmerged in all calculations because of the twinning. The final *R*_1_ was 0.1190 (*I* > 2σ­(*I*)) and w*R*_2_ was 0.3789 (all data). Twinning and whole-molecule disorder of both independent molecules in the asymmetric unit of this structure hampered solution, which was eventually achieved by recognizing the probably correct framework after fitting appropriate idealized rigid cluster fragments to selected observed electron density peaks; theoretical calculations optimizing the geometry of this S_2_B_17_ framework and identifying H atom positions, satisfactorily matching the observed NMR spectra for this and the Se analogue, provided a starting rigid-geometry model for fitting the major and minor disorder components, which was then relaxed with appropriate restraints on geometry and displacement parameters and constrained riding H atoms to give the final refinement. Relatively high crystallographic *R* factors and residual electron density indicate imperfect modeling of the disorder and/or twinning.

## Supplementary Material







## Notes

Experimental details, tables of NMR data and spectra, tables of Cartesian coordinates from DFT calculations (PDF)
Transition states TS1 (MP4)
Transition states TS2 (MP4)

## References

[1] b Housecroft, C. E. Boranes and Metallaboranes: Structure, Bonding and Reactivity; Halsted Press, 1994; Vol. 1994.

[2] Bould J., Londesborough M. G. S., Litecká M., Macías R., Shea S. L., McGrath T. D., Clegg W., Kennedy J. D. (2022). Macropolyhedral
Chalcogenaboranes: Insertion of Selenium
into the Isomers of B_18_H_22_. Inorg. Chem..

[3] Jelínek T., Kennedy J. D., Štíbr B., Thornton-Pett M. (1994). Macropolyhedral Boron-Containing Cluster Chemistry:
Isolation and Characterization of the First Macropolyhedral Thiaborane,
the *arachno*-Type [9,9′-S_2_B_17_H_18_]^−^ Ion. Angew. Chem., Int. Ed. Engl..

[4] Jemmis E. D., Balakrishnarajan M. M., Pancharatna P. D. (2001). A unifying electron-counting rule
for macropolyhedral boranes, metallaboranes, and metallocenes. J. Am. Chem. Soc..

[5] Grimes, R. N. Carboranes, 3rd ed.; Elsevier: Oxford, UK, 2016.

[6] Bould J., Tok O., Litecká M., Londesborough M. G. S., Ehn M. (2022). Two New Macropolyhedral Chalcogenaboranes:
S_2_B_16_H_18_ and SeB_16_H_18_. Inorg. Chim. Acta.

[7] Binder H., Ziegler A., Ahlrichs R., Schiffer H. (1987). μ_4_-S­(B_2_H_5_)_2_, (H_2_BSH)_2_, 1,2-(HS)_2_B_2_H_4_:
Neue Thiaborane aus der Reaktion zwischen Diboran und Schwefelwasserstoff
Theoretische Untersuchung der Molekülstrukturen. Chem. Ber..

[8] Bould J., Londesborough M. G. S., Tok O. (2024). Experimental and Computational ^77^Se NMR Spectroscopic Study on Selenaborane Cluster Compounds. Inorg. Chem..

[9] Ellis, P. D. ; Odom, J. D. ; Lipton, A. S. ; Chen, Q. ; Gulick, J. M. Nuclear Magnetic Shieldings and Molecular Structure; Tossell, J. A. , Ed.; Springer: Netherlands: Dordrecht, 1993; pp 539–555.

[10] Macgregor S. A., Welch A. J. (2023). Bridges and Vertices in Heteroboranes. Molecules.

[11] Bould J., Machacek J., Londesborough M. G. S., Macías R., Kennedy J. D., Bastl Z., Rupper P., Baše T. (2012). Decaborane Thiols as Building Blocks
for Self-Assembled Monolayers on Metal Surfaces. Inorg. Chem..

[12] Dosangh P. K., Bould J., Londesborough M. G. S., Jelínek T., Thornton-Pett M., Štíbr B., Kennedy J. D. (2003). Macropolyhedral
boron-containing cluster chemistry. Aspects of the S_2_B_16_H_16_ system. Preparation, structure, NMR spectroscopy
and isomerism. J. Organomet. Chem..

[13] Plażuk, D. ; Łomzik, M. ; Chrabąszcz, K. ; Wieczorek-Błauż, A. Thiiranes and Thiirenes—Monocyclic. In Reference Module in Chemistry, Molecular Sciences and Chemical Engineering; Elsevier, 2020.

[14] Olsen F. P., Vasavada R. C., Hawthorne M. F. (1968). The chemistry
of *n*-B_18_H_22_ and *i*-B_18_H_22_. J. Am. Chem.
Soc..

[15] Bondarev O., Sevryugina Y. V., Jalisatgi S. S., Hawthorne M. F. (2012). Acid-Induced
Opening of [*closo*-B_10_H_10_]^2–^ as a New Route to 6-Substituted *nido*-B_10_H_13_ Decaboranes and Related Carboranes. Inorg. Chem..

[16] Barton L., Bould J., Fang H., Hupp K., Rath N. P., Gloeckner C. (1997). A unique *nido exo-arachno* equilibrium
involving (PPh_3_)_2_(CO)­OsB_5_H_9_ and its base adducts: Crystal and molecular structure of {(PPh_3_)_2_(CO)­OsB_4_H_7_}­(BH_2_.PPh_2_Me). J. Am. Chem. Soc..

[17] Lott J. W., Gaines D. F. (1974). Manganese and rhenium
metalloboranes containing tridentate
borane ligands tridecahydrononaborate­(2-) and (tetrahydrofuran or
diethyl ether)­dodecahydrononaborate­(1-). Inorg.
Chem..

[18] Bould J., Crook J. E., Greenwood N. N., Kennedy J. D., Thornton-Pett M. (1990). Ten-vertex
metallaborane chemistry: synthesis and characterization of some *ortho*-cycloboronated *nido*-5- and −6-iridadecaboranes;
crystal structures of [5-H-5-(PPh_3_)-5-(PPh_2_-*o*-C_6_H_4_)-*nido*-5-IrB9H_12_-2] and [5-H-5,7-(PPh_3_)_2_-5-(PPh_2_-*o*-C_6_H_4_)-*nido*-5-IrB_9_H_12_-2]. J. Chem.
Soc., Dalton Trans..

[19] Štíbr B., Holub J., Bakardjiev M., Lane P. D., Mckee M. L., Wann D. A., Hnyk D. (2017). Unusual Cage
Rearrangements in 10-Vertex *nido*-5,6-Dicarbaborane
Derivatives: An Interplay between
Theory and Experiment. Inorg. Chem..

[20] Frisch, M. J. ; Trucks, G. W. ; Schlegel, H. B. ; Scuseria, G. E. ; Robb, M. A. ; Cheeseman, J. R. ; Scalmani, G. ; Barone, V. ; Petersson, G. A. ; Nakatsuji, H. Gaussian 16, Revision C.01; Gaussian Inc.: Wallingford CT, 2016.

[21] Pritchard B. P., Altarawy D., Didier B., Gibson T. D., Windus T. L. (2019). New Basis
Set Exchange: An Open, Up-to-Date Resource for the Molecular Sciences
Community. J. Chem. Inf. Model..

[22] Peng C., Schlegel H. B. (1993). Combining Synchronous Transit and Quasi-Newton Methods
to Find Transition States. Isr. J. Chem..

[23] Oxford Diffraction . CrysAlisPRO, Version 1.0.43; Agilent Technologies UK Ltd: Yarnton, UK, 2020.

[24] Coppens P., Row T. N. G., Leung P., Stevens E. D., Becker P. J., Yang Y. W. (1979). Net atomic charges
and molecular dipole moments from
spherical-atom X-ray refinements, and the relation between atomic
charge and shape. Acta Crystallogr., Sect. A.

[25] North A. C. T., Phillips D. C., Mathews F. S. (1968). A semi-empirical method of absorption
correction. Acta Crystallogr., Sect. A.

[26] Sheldrick G. M. (2015). SHELXT
- Integrated space-group and crystal-structure determination. Acta Crystallogr., Sect. A:Found. Adv..

[27] Sheldrick G. M. (2015). Crystal
structure refinement with SHELXL. Acta Crystallogr.,
Sect. C:Struct. Chem..

[28] Dolomanov O. V., Bourhis L. J., Gildea R. J., Howard J. A. K., Puschmann H. (2009). OLEX2: a complete
structure solution, refinement and analysis program. J. Appl. Crystallogr..

