# Supplementary material for: Proton-Initiated Reversible Chalcogen-Vertex Extrusion in Macropolyhedral Chalcogenaboranes
Source: Inorg Chem. 2026 Feb 18;65(8):4382–9. doi: 10.1021/acs.inorgchem.5c03815 (PMC12958281; doi:10.1021/acs.inorgchem.5c03815)
Supplement: Supplementary file 1 [file ic5c03815_si_001.pdf]

Supporting Information

Proton-Initiated Reversible Chalcogen-Vertex Extrusion in Macropolyhedral Chalcogenaboranes

Jonathan Bould,*[,a] Miroslava Litecká,[a] William Clegg,[b] Marcel Ehn,[a] John D. Kennedy,[a,c] and Michael G. S. Londesborough*[,a]

[a] Institute of Inorganic Chemistry of the Czech Academy of Sciences, 250 68 Husinec-Řež č.p. 1001, Czech Republic

[b] School of Natural and Environmental Sciences, Newcastle University Newcastle upon Tyne, NE1 7RU, UK

[c] School of Chemistry, University of Leeds, Leeds, LS2 9JT, UK

Email: bould@iic.cas.cz, michaell@iic.cas.cz

Table of Contents

		Page
**Table S1**	Measured boron-11 and proton NMR data for $S_2B_{17}H_{19}$ **2a**.	S2
**Table S2**	Measured boron-11 and proton NMR data for $Se_2B_{17}H_{19}$ **2a**.	S3
**Figure S1**	Comparison line drawings of measured and calculated NMR spectrum of $S_2B_{17}H_{19}$ **2a**.	S4
**Figure S2**	^{11}B-{^1H} NMR spectra for $S_2B_{17}H_{19}$ **2a** and $Se_2B_{17}H_{19}$ **2b**.	S4
**Figure S3**	Measured and calculated ^{77}Se NMR spectra for $Se_2B_{17}H_{19}$ **2b**.	S5
**Table S3**	Selected Interatomic distances for $Se_2B_{17}H_{19}$ **2a**.	S6
**Tables S4 to S14**	DFT calculated Cartesian atomic coordinates.	S7 to S17
	References	S18

Table S1. Measured[1] boron-11 and proton NMR data for $S_2B_{17}H_{19}$ **2a** in CD_2Cl_2 solution, 192 MHz, 294 K together with DFT/GIAO calculated ^{11}B chemical shifts.

Assignments	Calculated	Measured	
	$\delta(^{11}B)$/ppm	$\delta(^{11}B)$/ppm	$\delta(^{1}H)$/ppm
10	+28.7	+25.6	+5.16
8	+15.9	+13.4	+4.25
1′	+11.3	+11.9	+3.82
6[a]	+9.2	+10.3	---
7′	+7.2	+8.0	+4.16
3	+6.8	+6.5	+3.62
9′	+4.5	+4.1	+3.68
3′	–3.1	–1.9	+2.77
5[a]	–1.6	–3.3	---
1	–6.4	–6.0	+2.89
10′	–7.8	–10.8	+2.82
8′	–9.1	–12.3	+2.84
2	–21.5	–19.8	+1.70
7	–26.8	–28.4	+1.86
4	–30.0	–30.7	+0.74
2′	–37.6	–38.0	–0.77
4′	–44.5	–44.1	+0.54
μH-(9′,10′)	–0.13		–0.24
μH-(5,7′)	–0.81		–1.17
μH-(6,7)	–1.42		–1.60
S–H	+1.60		[b]

[a] Site of commo boron atom linking the two subclusters and holding no terminal hydrogen atom. [b] The thiol hydrogen resonance was not located.

Table S2. Measured[1] boron-11 and proton NMR data for $Se_2B_{17}H_{19}$ **2b** in CD_2Cl_2 solution, 192 MHz, 263 K together with DFT/GIAO calculated ^{11}B chemical shifts.

Assignments	Calculated	Measured	
	δ(^{11}B)/ppm	δ(^{11}B)/ppm	δ(^{1}H)/ppm
10	+33.7	+30.3	+5.95
8	+20.6	+17.7	+5.01
1′	+10.9	+11.9	+3.95
6[a]	+7.7	+10.2	---
7′	+7.1	+7.2	+4.11
3	+7.6	+6.4	+4.09
9′	+6.3	+4.6	+3.91
3′	–3.8	–2.3	+3.02
5[a]	–2.5	–2.7	---
1	–6.9	–5.8	+3.32
8′	–6.6	–11.0	+2.92
10′	–9.8	–11.7	+3.00
2	–20.9	–18.2	+2.19
7	–26.9	–27.6	+1.95
4	–27.2	–28.3	+0.65
2′	–36.2	–36.2	–0.70
4′	–43.2	–43.2	+0.74
μH-(9′,10′)	–0.03		–0.23
μH-(5,7′)	–1.08		–1.35
μH-(6,7)	–1.40		–1.60
Se–H	+1.30		[b]

[a] Site of commo boron atom linking the two sublcusters and holding no terminal hydrogen atom. [b] The selenol hydrogen resonance was not located.

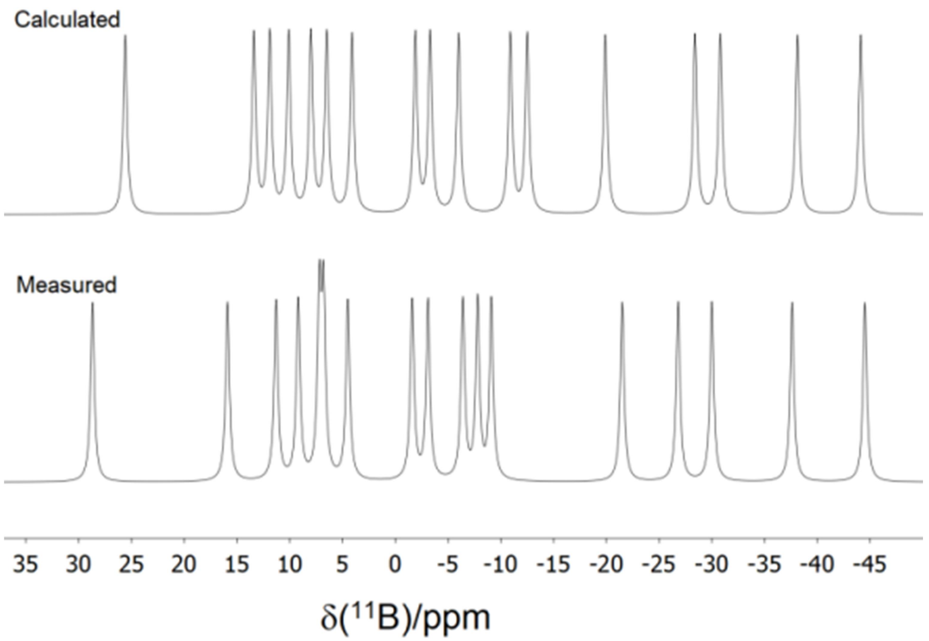

Figure S1. An illustration of the relative positions of the measured ^{11}B-{^{1}H} resonances of the eighteen-vertex $S_2B_{17}H_{19}$ **2a** together with its DFT/GIAO-calculated chemical shifts.

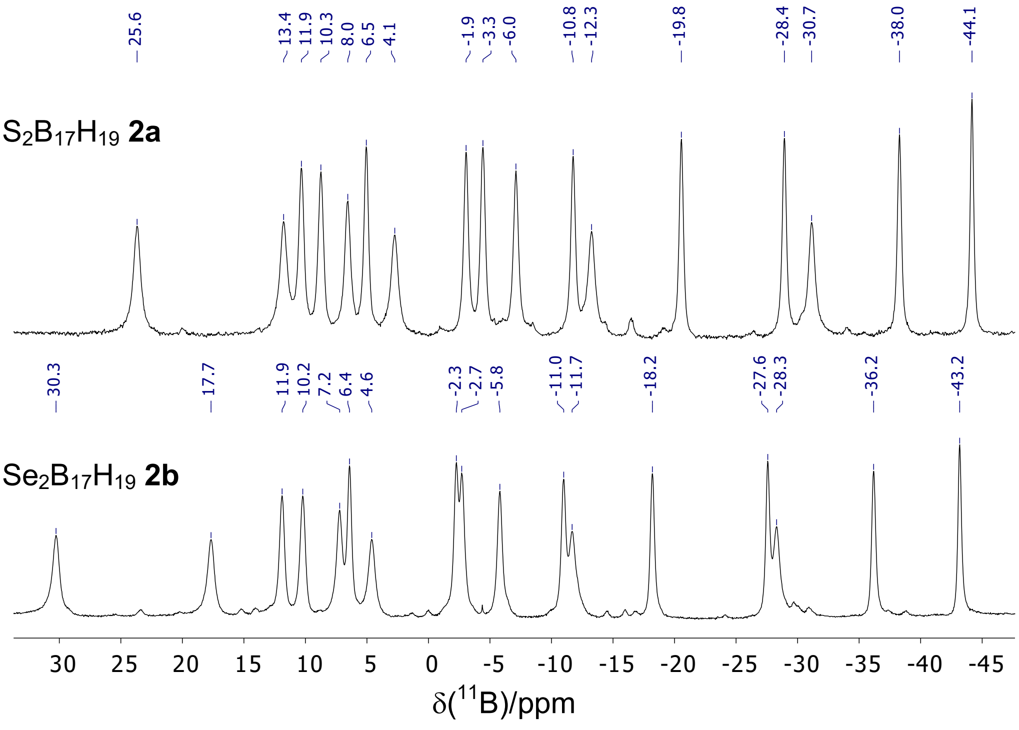

Figure S2. 192 MHz, ^{11}B-{^{1}H} NMR spectra in CD_2Cl_2 for $S_2B_{17}H_{19}$ **2a** 294 K and $Se_2B_{17}H_{19}$ **2b** 263 K.

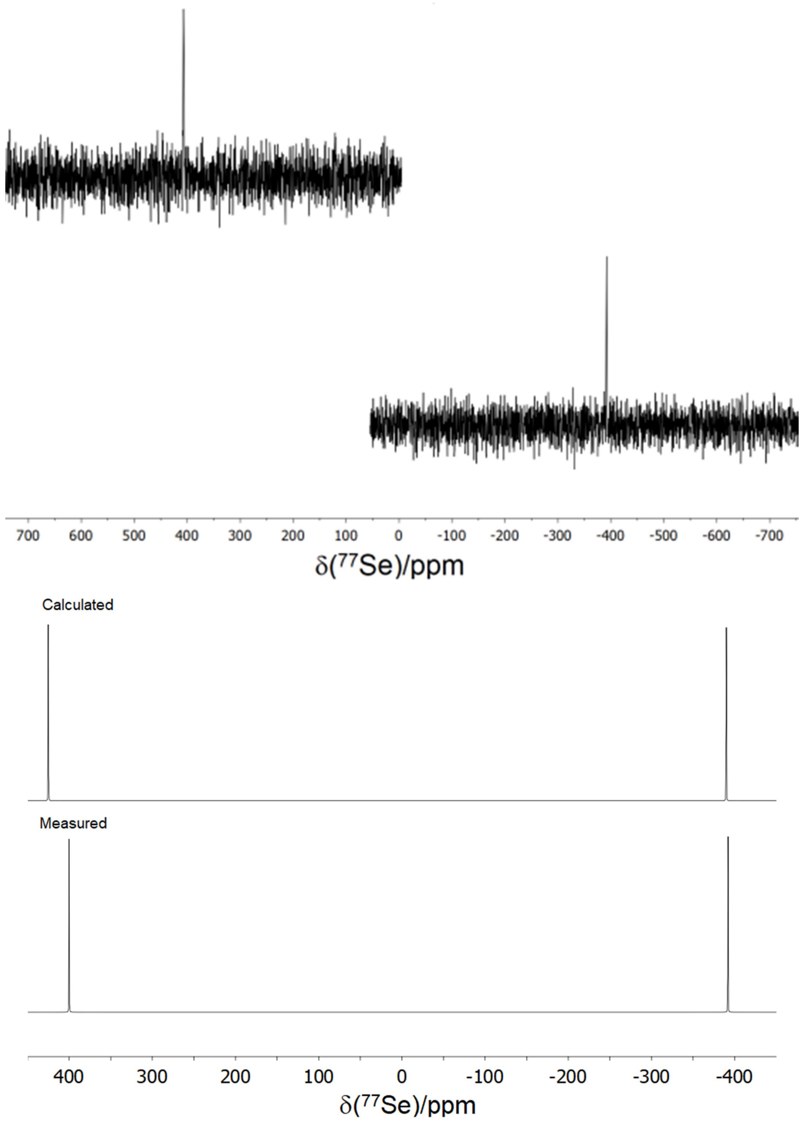

Figure S3. (Upper) 114.5 MHz ^{77}Se NMR spectra for $Se_2B_{17}H_{19}$ **2b**. CD_2Cl_2 solution at 253 K. The two regions of the spectra were measured separately. (Lower) A plot of the calculated versus measured ^{77}Se chemical shifts.

Table S3. Selected Interatomic distances (Å) for for $S_2B_{17}H_{19}$ **2a**.

B1–H1	1.150	B1–B2	1.826(9)
B1–B3	1.842(9)	B1–B4	1.746(8)
B1–B5	1.787(8)	B1–B10	1.709(10)
B2–H2	1.150	B2–B3	1.776(8)
B2–B5	1.822(9)	B2–B6	1.763(8)
B2–B7	1.786(7)	B3–H3	1.1501
B3–B4	1.759(9)	B3–B7	1.782(8)
B3–B8	1.729(8)	B4–H4	1.150
B4–B8	1.878(7)	B4–S9	1.991(7)
B4–B10	1.871(9)	B5–B6	1.800(7)
B5–B10	1.905(9)	B5–B2'	1.752(8)
B5–B7'	1.833(9)	B5–H57'	1.2727
B6–B7	1.802(8)	B6–H67	1.4037
B6–B1'	1.746(7)	B6–B2'	1.820(9)
B6–B10'	1.903(9)	B7–H7	1.150
B7–H67	1.2831	B7–B8	1.908(9)
B8–H8	1.150	B8–S9	1.856(7)
S9–B10	1.849(7)	B10–H10	1.150
B1'–H1'	1.1501	B1'–B2'	1.784(8)
B1'–B3'	1.778(8)	B1'–B4'	1.785(8)
B1'–B10'	1.716(9)	B2'–H2'	1.150
B2'–B3'	1.786(8)	B2'–B7'	1.772(8)
B3'–H3'	1.150	B3'–B4'	1.774(9)
B3'–B7'	1.709(8)	B3'–B8'	1.766(9)
B4'–H4'	1.1499	B4'–B8'	1.823(8)
B4'–B9'	1.758(9)	B4'–B10'	1.775(9)
B7'–H7'	1.150	B7'–H57'	1.3187
B7'–B8'	2.039(10)	B8'–H8'	1.150
B8'–B9'	2.058(10)	B8'–S11'	2.006(7)
B9'–H9'	1.1499	B9'–B10'	1.823(9)
B9'–H91'	1.3415	B9'–S11'	1.914(7)
B10'–H10'	1.1501	B10'–H91'	1.2935
S11'–H11'	1.3483		

Table S4. $Se_2B_{17}H_{19}$ **2b**. DFT/mPW1PW91/6-31+G(d,p) calculation for ^{77}Se shieldings with IEFPCM model for CH_2Cl_2 solvent. ZPE = -5236.790923 H.

B -1.4537319428 2.0466885555 -0.9152043205
B 0.2082988824 1.4952391571 -0.6488080595
B -0.7202502458 2.1855924485 0.6991891741
B -0.9284759039 0.5763789466 -1.6700341693
B -2.8005550379 0.9090987498 -0.8956593416
B 1.9528204453 -0.5860535132 -1.3585108891
B -2.4744634508 1.9865891875 0.5353482982
B 0.2511898056 -0.2495765655 -0.5614324808
B 0.122037238 0.6779622926 0.9748391448
B 2.9232108576 -1.574726151 -0.112703061
B 3.0675417969 -0.398914909 1.3297128661
B -1.5684954612 1.1100268301 1.776136333
B -3.0509735341 0.4141766211 1.0191827384
B 1.1656952676 -1.7457926934 -0.3628072098
B 1.3426882807 -0.1243643085 2.0350859948
B 0.2305840432 -1.0786661881 1.0300877268
B 1.8764904695 -1.6437884974 1.3059901263
Se 3.4338498706 0.4131451932 -0.4788109681
Se -3.160600901 -1.1233917505 -0.416641316
H -3.6962769487 1.2368366225 -1.6030704056
H -4.0407996865 0.3662755021 1.6704471043
H -3.1723697128 2.922863627 0.7617653536
H -1.6234190179 1.3686528395 2.9345759098
H -0.3041429327 3.1732193649 1.2139962672
H -1.6288574313 2.9490135274 -1.6700721916
H -0.8502011557 0.437375827 -2.8456766884
H 1.1019686104 2.1380564926 -1.0851980705
H 1.2148920842 -0.023279753 3.2112964848
H -0.662396052 -1.7435023111 1.4448067962
H 0.722430178 -2.722283638 -0.8772073843
H 1.9975354831 -0.6315303141 -2.5424103246
H 3.7083807143 -2.4187236848 -0.3868093925
H 1.9891735956 -2.5578580469 2.0587683654
H 4.0004844616 -0.3574007471 2.0623970703
H 1.3515149372 1.0462861641 1.4952081756
H -1.9768172519 -0.119789405 1.664132874
H -0.8339117984 -0.6519398671 -1.1890937616
H -4.6227499764 -1.0103791752 -0.463480089

Table S5. DFT/mPW1PW91/6-31+G(d,p) calculation of ^{77}Se shieldings of $Se_2B_{17}H_{19}$ with *endo* Se-H. IEFPCM model for CH_2Cl_2 solvent. ZPE = -5236.786431 H.

B	-1.6826933812	0.5285767504	-1.9066209921
B	0.0353278114	0.3019149703	-1.5423567022
B	-0.8022421284	1.7607965512	-0.9710003719
B	-1.1231303685	-1.053347284	-1.46800012
B	-2.910209937	-0.2360072685	-0.9018450063
B	1.8510265479	-1.6994598417	-0.7868409879
B	-2.5425834835	1.5448225267	-0.7375950159
B	0.2180283705	-0.8707239523	-0.2613900376
B	0.1673849672	0.8757228952	0.1806274522
B	3.0108404959	-1.532794118	0.663190645
B	3.2087089108	0.3156816522	0.8242307353
B	-1.4632076737	1.7897780077	0.6407490936
B	-2.9951853911	0.8338824094	0.7890788199
B	1.2545242762	-1.7996706367	0.8228781918
B	1.5440899833	1.0405936629	1.3354494149
B	0.4146909319	-0.3251581999	1.4399430974
B	2.1178563099	-0.5577732481	1.8306170789
Se	3.3329719333	-0.3972520506	-1.0560022527
Se	-3.2757316648	-1.2493296678	0.9174157427
H	-3.9060098546	-0.4914028511	-1.5008985295
H	-3.9030774357	1.3506772675	1.3520101801
H	-3.2764923112	2.3732020456	-1.1729514324
H	-1.4057034258	2.7927418568	1.275163801
H	-0.4052405918	2.8130709494	-1.3568660348
H	-2.0092700143	0.6244708123	-3.0461043491
H	-1.1499645228	-1.9787647483	-2.2098986415
H	0.831265651	0.4363208362	-2.4086989387
H	1.5264828163	1.9462788223	2.1028696749
H	-0.3686623051	-0.4738820709	2.3227227798
H	0.8361166482	-2.8454261583	1.2061160713
H	1.7832401941	-2.5719224679	-1.5866764079
H	3.8255731398	-2.336061319	0.9708715263
H	2.3700119474	-0.6709496558	2.9873901234
H	4.2013324277	0.8513869797	1.1934100881
H	1.4104821116	1.4838062645	0.1322208784
H	-1.8362853267	0.8571402945	1.4891436389
H	-0.8911752504	-1.5785173595	-0.2868871811
H	-1.9376935879	-1.5731592163	1.4278794283

Table S6. $Se_2B_{17}H_{19}$, **2b**. DFT/B3LYP/6-31+G(d,p) (Binning 962(d) for Se) calculation for ^{11}B shieldings with IEFPCM model for CH_2Cl_2 solvent. ZPE = -5236.553752 H.

B -1.4957305667 2.0092447028 -0.9647309032
B 0.1775174733 1.4803125581 -0.6918031414
B -0.7413997393 2.2188363881 0.6416815801
B -0.9589256818 0.5185205387 -1.6724272238
B -2.857820845 0.8911985869 -0.8804810983
B 1.954292305 -0.6489941184 -1.3432038354
B -2.504963656 2.0177932294 0.5060698597
B 0.2402755766 -0.262168293 -0.5367664108
B 0.1221939198 0.7321386283 0.9692137854
B 2.9277832183 -1.5830632241 -0.052972184
B 3.0994584495 -0.339927811 1.3373960479
B -1.5814467391 1.1998927562 1.781794868
B -3.0884345118 0.4770839172 1.0783343532
B 1.1646454396 -1.748008225 -0.284979713
B 1.3697523822 -0.0225659506 2.0526180339
B 0.2436710454 -1.0202335964 1.0960463872
B 1.8973071983 -1.5743809294 1.3803286195
Se 3.455155932 0.4011722919 -0.5228546149
Se -3.2369998725 -1.142587155 -0.3049944144
H -3.7593570195 1.1854318401 -1.59036413
H -4.0657741531 0.4607411891 1.7456113074
H -3.1883664436 2.9720303472 0.6935872444
H -1.6183497358 1.5134304335 2.9257474418
H -0.3246201589 3.2303391864 1.1040828913
H -1.675425147 2.8869483728 -1.7455689755
H -0.8929446624 0.3323894364 -2.8402080214
H 1.0610394711 2.1138161241 -1.156013049
H 1.2535931445 0.121419609 3.2241485261
H -0.6394093187 -1.6707254013 1.5452303463
H 0.71599912 -2.7477453148 -0.7440280265
H 1.9858756364 -0.7483137403 -2.522030667
H 3.706520347 -2.4395219699 -0.2966931675
H 2.0128342417 -2.455841864 2.1690207348
H 4.0374711641 -0.2687053122 2.0579506173
H 1.3469244066 1.1245166867 1.4725610707
H -2.0121881586 -0.0253612857 1.7334662117
H -0.8613684102 -0.6782472896 -1.1292220569
H -4.7020726513 -1.009530342 -0.3472182937

Table S7. $Se_2B_{17}H_{19}$ with *endo* Se-H. DFT/B3LYP/6-31+G(d,p) (Binning 962(d) for Se) with IEFPCM model for CH_2Cl_2 solvent. ZPE = -5236.549436 H.

B -1.6939065382 0.5222368839 -1.9054216266
B 0.029181178 0.3016843319 -1.5453918305
B -0.8106979638 1.7596640326 -0.9639706082
B -1.1229577095 -1.0603949388 -1.4778704813
B -2.9431928229 -0.2288439097 -0.9160322981
B 1.8849893823 -1.7206720962 -0.7881238777
B -2.5601051375 1.5476299089 -0.735642869
B 0.2294864453 -0.8756603139 -0.2676611471
B 0.1751326215 0.87819589 0.1785710958
B 3.0340727309 -1.5432129126 0.6734292966
B 3.2436041772 0.3107816997 0.8293844393
B -1.4787642388 1.8016291528 0.6466185625
B -3.0243128951 0.853400625 0.7957071757
B 1.2731606179 -1.8041018121 0.8152428295
B 1.5650827598 1.047337313 1.3347298322
B 0.4319949541 -0.3231153044 1.4362169919
B 2.1398631594 -0.5543833644 1.8300663994
Se 3.3762871262 -0.4070993292 -1.0673819767
Se -3.3408349445 -1.2547196267 0.923607432
H -3.934269659 -0.4835100091 -1.5171601202
H -3.9270892991 1.3715224162 1.3609582759
H -3.2807931682 2.3799427285 -1.183125711
H -1.4185652237 2.8061645414 1.2752716339
H -0.418354546 2.8104526663 -1.3548511601
H -2.0136043917 0.6300146411 -3.0447609425
H -1.1480492392 -1.9839939707 -2.2189919514
H 0.8203774006 0.4469162713 -2.4115319204
H 1.5462771041 1.9483255437 2.1055381576
H -0.3502122059 -0.4805976231 2.313791766
H 0.8561837954 -2.8458787657 1.2063455825
H 1.8178287731 -2.593843087 -1.5840059887
H 3.8454954445 -2.3455938707 0.9847975785
H 2.3860488858 -0.6687047344 2.9869622823
H 4.2334526647 0.8457672785 1.2006401925
H 1.4081767858 1.497389811 0.1405659107
H -1.8713905445 0.8780839066 1.494935699
H -0.890838397 -1.5693460476 -0.2933948618
H -2.0133602619 -1.614204486 1.4452836977

Table S8. $S_2B_{17}H_{19}$ **2a**. DFT/B3LYP/6-31+G(d,p) calculation for ^{11}B shieldings with IEFPCM model for CH_2Cl_2 solvent. ZPE = -1230.245472 H

B -1.4997862628 1.9877699721 -0.9690209805
B 0.17803523 1.4666086012 -0.688213671
B -0.7508331706 2.2070026925 0.6401394198
B -0.9418755802 0.5150331833 -1.6896338672
B -2.8504816972 0.8618042352 -0.9103444404
B 1.951242036 -0.6210731938 -1.3147232959
B -2.5165746968 1.9993865654 0.495289437
B 0.2247553903 -0.2763147277 -0.5357162541
B 0.1074356693 0.7165882146 0.9702251504
B 2.9226235365 -1.5819495907 -0.0612864261
B 3.0711451415 -0.3248763337 1.2974597586
B -1.5962163355 1.2043436311 1.7874944097
B -3.0999525696 0.4609215321 1.1088568017
B 1.1574214658 -1.7574079589 -0.2922138847
B 1.3542317658 -0.0361245389 2.0474277785
B 0.2312517716 -1.0394404754 1.092151065
B 1.8914840936 -1.5857008987 1.3739336915
S 3.3403326619 0.2976592669 -0.4699897657
S -3.1741797886 -0.9776675938 -0.2301252142
H -3.7494663477 1.1469471368 -1.6283633437
H -4.0787487535 0.4558937942 1.7760641621
H -3.1890663659 2.9628210618 0.6753723573
H -1.638963916 1.558934718 2.9193713936
H -0.3290229797 3.2177130349 1.0989324539
H -1.6867938604 2.8679031331 -1.7453678676
H -0.8540209388 0.3397426846 -2.8576172105
H 1.070721454 2.1007881527 -1.1352372732
H 1.2519865364 0.1041759898 3.2203246989
H -0.6501353253 -1.6875941857 1.546882081
H 0.715037315 -2.7536663653 -0.7632665096
H 2.0120540455 -0.6809678269 -2.4948452492
H 3.7172437646 -2.4193179556 -0.3174585345
H 2.0168945175 -2.4637823375 2.1639482144
H 4.0248220828 -0.2164314108 1.9922570315
H 1.3385878552 1.1105266072 1.4721786692
H -2.0202420796 -0.0177919261 1.7950721519
H -0.8656489917 -0.6873258497 -1.1534591021
H -4.5185836735 -0.9537960384 -0.3281678358

Table S9. DFT/B3LYP/6-31+G(d,p) calculation for $[S_2B_{17}H_{18}]^-$ anion **2a**$^-$ after deprotonation of the μ^2-{SH} proton in $S_2B_{17}H_{19}$ **2a** with IEFPCM model for CH_2Cl_2 solvent ZPE = -1229.825701 H.

B 1.8040827895 -1.7147264279 -0.7882082637
B 0.0447115982 -1.4787102048 -0.5523885608
B 1.0966177498 -1.8010904844 0.8575583856
B 0.9986325083 -0.51419754 -1.7158436467
B 2.9466548448 -0.3449459333 -0.9331246154
B -2.0284810004 0.0840702527 -1.516402831
B 2.7746024767 -1.2961927677 0.6472189162
B -0.3067008402 0.223472669 -0.698730775
B -0.0370809936 -0.4691061823 0.9510179711
B -3.2269451794 1.0206266657 -0.440547485
B -3.1671151541 0.0022797092 1.110471803
B 1.7055130173 -0.4649162065 1.7960822841
B 3.099689298 0.4385938846 0.9310991458
B -1.5208097987 1.5099729917 -0.6964869379
B -1.4691381596 0.176697872 1.8881148419
B -0.5216182675 1.2225754247 0.7937601797
B -2.2603744456 1.4694119634 0.9680109291
S -3.2602845931 -0.9380095062 -0.5413372353
S 2.9834206697 1.5499796194 -0.5822144459
H 3.8701328088 -0.7054342551 -1.6005730914
H 4.020064846 0.6270544304 1.6701478868
H 3.5964963926 -2.0921927859 0.9863077941
H 1.7976422326 -0.6332489381 2.9726647585
H 0.8597572153 -2.7907508817 1.4764033756
H 2.1418076444 -2.6817418831 -1.3984511603
H 0.8779466163 -0.5276066323 -2.896960648
H -0.7148964047 -2.3345492047 -0.8604979776
H -1.3777887074 0.2592478835 3.0699065948
H 0.193231892 2.1005666245 1.1412654739
H -1.2743756681 2.4891731304 -1.3239941551
H -2.0731874275 -0.0525078659 -2.6922843612
H -4.1608676427 1.6342565011 -0.8329667952
H -2.5892046193 2.4254147994 1.5953996233
H -4.1212517413 -0.1623836016 1.7974507769
H -1.1960626527 -1.0118657538 1.5020879239
H 1.8792785297 0.7677148759 1.5656730593
H 0.7192181658 0.7272357577 -1.3271897381

Table S10. DFT/B3LYP/6-31+G(d,p) calculation for $[S_2B_{17}H_{18}]^-$ anion **1a** with IEFPCM model for CH_2Cl_2 solvent. ZPE = -1229.840529 H.

B -1.7666346175 2.0351907383 -0.4471659484
B -0.2142422162 1.0782620443 -0.6123365458
B -0.9777876351 1.4334369278 1.027453687
B -1.725369588 0.9374111661 -1.7656569909
B -3.2936840598 1.2456347778 -0.7827021026
B 2.0206241452 -0.374475131 -1.5830055233
B -2.7518264231 1.3163873656 0.9034760346
B 0.3379281627 -0.7301029817 -0.7609935415
B -0.0670196301 0.0127001959 0.7975217906
B 3.4039690193 -0.9142675575 -0.435960818
B 3.0786791883 0.368606019 0.9803240405
B -1.8111664185 -0.0260233735 1.550617235
B -3.1907497509 -0.3156388359 0.3615275554
B 1.8569790309 -1.7288212266 -0.5417628244
B 1.5222979144 -0.1424093259 1.8274459375
B 0.8174296536 -1.4409781801 0.8708245772
B 2.584168901 -1.3330574043 1.0787554624
S 3.1332598718 0.9748020853 -0.8472949534
S -2.9219183252 -0.516888179 -1.5352850206
H -4.2844236255 1.7944797874 -1.1378205016
H -4.1565337628 -0.889406643 0.7473630921
H -3.4118926327 1.9890206884 1.631247391
H -1.9667160932 -0.3514591672 2.6849175349
H -0.5591135344 2.1859125265 1.8512854005
H -1.6592387896 3.2044788998 -0.6527425177
H -1.5884045779 1.2533602108 -2.9038949917
H 0.6019004211 1.8745543826 -0.940838407
H 1.4762763737 -0.0693804086 3.0145790957
H 0.2785351229 -2.3439922375 1.4315084612
H 1.8078466253 -2.8251393766 -1.007273628
H 2.0854299733 -0.4377343828 -2.7687394381
H 4.4507114805 -1.3930017608 -0.7258750804
H 3.122926284 -2.1575728958 1.7478503704
H 3.9538341054 0.8288418501 1.6384309753
H 1.8252314222 0.9815461958 1.3397762496
H -2.0057167586 -1.0142728335 0.7901124994
H -0.395391147 -1.4227437201 -1.3859534366

Table S11. DFT/B3LYP/6-31+G(d,p) calculation for $[S_2B_{17}H_{18}]^-$ anion **1a'** with IEFPCM model for CH_2Cl_2 solvent. ZPE = -1230.214352H.

B 2.0058542761 -0.3558570528 1.665994544
B 0.3645590585 -0.5375402154 0.8760749183
B 1.0482145301 1.11259053 1.3243188384
B 1.9797090704 -1.5617897135 0.4501035423
B 3.466111613 -0.4481866402 0.6916145261
B -1.9305445694 -1.5829341648 -0.5369980214
B 2.7913363014 1.1547193249 0.9930035441
B -0.4125323374 -0.5760608318 -0.8898073386
B 0.0145039937 0.9263523743 -0.0052213031
B -3.4007688986 -0.4511708736 -0.6639318042
B -3.1328725688 0.8907866812 0.7096054803
B 1.643873761 1.8251026355 -0.1704582552
B 3.0788234078 0.7951774461 -0.7266391442
B -2.020867891 -0.3073715648 -1.6873970477
B -1.7047352478 1.8648947155 0.1534344745
B -1.063893314 1.1249529351 -1.3046193546
B -2.832758604 1.1636503619 -0.9967609347
S -2.7221844171 -1.0811716625 1.206486421
S 2.9476670989 -1.0909609781 -1.0865106957
H 4.5388077944 -0.757287335 1.0870461709
H 3.9255751453 1.3240974315 -1.3654709308
H 3.462063144 1.8691932908 1.6647834293
H 1.6647640775 2.987356914 -0.4137371679
H 0.6551897006 1.821987121 2.194387337
H 2.0325518554 -0.6973376719 2.8056912067
H 1.9197847853 -2.7288325597 0.6542237025
H -0.3106410877 -1.0414949441 1.7044893162
H -1.6780546341 3.0184216707 0.4228935789
H -0.6745993532 1.8448844072 -2.1653274227
H -2.1167411375 -0.6321203252 -2.8256234667
H -2.0150983289 -2.7445605287 -0.7417331837
H -4.4986993643 -0.8522327312 -0.8268202905
H -3.5547915724 1.8333268393 -1.658905242
H -4.0269491443 1.2953371731 1.3656782916
H -1.946558217 1.2467361173 1.2761409515
H 1.7770987891 1.1483061386 -1.2304308385
H 0.2965826894 -1.0300478186 -1.7238420239
H -3.9089926643 -1.724001876 1.2983664425

Table S12. DFT/B3LYP/6-31+G(d,p) calculation for TS1 with IEFPCM model for CH_2Cl_2 solvent. ZPE = -1229.812354 H.

B -1.7122037098 1.8287486042 -0.6127721926
B -0.0119644516 1.4043066193 -0.3572222388
B -1.0469761745 1.6328397427 1.0463801861
B -1.2519348082 0.4140703271 -1.615239555
B -3.0785586531 0.7657648556 -0.8826048952
B 1.9557280201 0.0198183155 -1.5445680336
B -2.7708791851 1.2782854008 0.7762457804
B 0.344669536 -0.2620356388 -0.7015984702
B -0.0110983043 0.2607818581 0.9999664095
B 3.2759354931 -0.8829507976 -0.5613545149
B 3.1527955447 0.1614482912 1.0549851477
B -1.7517991849 0.1450188846 1.6913453854
B -3.1355688183 -0.437961022 0.5801323288
B 1.6364409021 -1.4880294982 -0.753964238
B 1.5408821599 -0.2363176313 1.871817105
B 0.6596700095 -1.304501214 0.7521605845
B 2.4167864603 -1.4464026522 0.8928771105
S 3.2724051262 1.060775808 -0.6506263103
S -2.8653000941 -1.1305554431 -1.2163041686
H -4.0113119459 1.2412800813 -1.4505531375
H -4.1288966557 -0.8448877714 1.0929528448
H -3.551299997 2.0271059148 1.2768873065
H -1.9059418785 0.0123244934 2.8664092559
H -0.8287099766 2.5187635023 1.813746035
H -1.8910212079 2.8980655724 -1.1101635982
H -1.1475324577 0.5222691622 -2.791656947
H 0.7487148476 2.2765054386 -0.6160130217
H 1.5629289524 -0.3765660072 3.0537711533
H 0.0307293365 -2.2303213634 1.1523805588
H 1.4433566731 -2.4852796521 -1.3738643253
H 1.9646117242 0.1523236415 -2.7252844895
H 4.2404965781 -1.4517141797 -0.9531521685
H 2.8596260915 -2.4284063102 1.3984599298
H 4.103842756 0.3528210328 1.7394162077
H 2.021436344 0.8872580167 1.548349807
H -1.9819724468 -0.9794895211 1.1405915914
H -0.5378884957 -0.7878966207 -1.3922173034

Table S13. DFT/B3LYP/6-31+G(d,p) calculation for TS2 with IEFPCM model for CH_2Cl_2 solvent. ZPE = -1230.209308 H.

B 1.9908992821 -0.1485512389 1.7210320549
B 0.3161983059 -0.3973970825 1.0696174292
B 1.0687669254 1.2835571085 1.2591313985
B 1.7016632642 -1.5218549113 0.8087430036
B 3.2774635235 -0.5004641014 0.6217261623
B -1.9708646458 -1.5685795724 -0.1829769364
B 2.8360451907 1.1981589635 0.9044737178
B -0.3391222435 -0.6098153361 -0.6880842986
B -0.0214634509 0.9881973728 -0.0155419606
B -3.4487268337 -0.5077860082 -0.6338986056
B -3.1118083012 0.9250492504 0.6097368544
B 1.7010083174 1.8853273341 -0.2543642218
B 3.1031876864 0.8554851503 -0.8076691612
B -1.9515341297 -0.5483600888 -1.5569556777
B -1.6550219553 1.8773816203 -0.0281136637
B -1.0084275672 0.9649782819 -1.3912164995
B -2.7576059584 1.0405952848 -1.1310364702
S -3.004750176 -0.8922669927 1.2318801121
S 2.8279571181 -1.0573966517 -1.2807581804
H 4.3491408579 -0.9192736745 0.8886776753
H 4.0191178038 1.3123256028 -1.3989895527
H 3.5886634732 1.8740576243 1.5242422735
H 1.7228411835 3.034019883 -0.5469573031
H 0.7274984764 2.086194 2.0649394703
H 2.1699322919 -0.4313738614 2.8631876432
H 1.736755868 -2.697163966 0.8340535273
H -0.4115849724 -0.6981846513 1.9552914343
H -1.696654687 3.0621571075 0.0442456255
H -0.588364052 1.5431285289 -2.3416731421
H -1.9363332114 -1.0386623827 -2.6405721688
H -1.9030236818 -2.7524747071 -0.2165731149
H -4.5053277547 -0.8696741735 -1.0279864314
H -3.4020684111 1.6490622013 -1.9222805897
H -3.9842779252 1.534927331 1.1310201586
H -1.8283192637 1.3537839118 1.1098853411
H 1.893861151 1.2244252408 -1.3504083392
H 0.3563518885 -1.1944313824 -1.4421468095
H 4.1091436135 -1.4585810149 -1.4334847546

Table S14. DFT/B3LYP/6-31+G(d,p) calculation of the transition state for $SB_{17}H_{18}$-SH_{exo} to $SB_{17}H_{18}$-SH_{endo}.

Transition state, ZPE = -1230.222012 H					$SB_{17}H_{18}$-SH_{exo} ZPE = -1230.239881 H			
B	-1.7320454159	1.8101738398	-0.620243639		B	-1.8153552112	1.7764389762	-0.688274428
B	-0.0372310603	1.3993215383	-0.3506296006		B	-0.0670332732	1.529830077	-0.4856630127
B	-1.0549336785	1.6400495343	1.0668308375		B	-1.0984479546	1.7577123577	0.9491772354
B	-0.9704695444	0.6583260326	-1.6764420318		B	-0.9997097483	0.6367916941	-1.7081594929
B	-2.9371586234	0.5618156607	-0.939146007		B	-2.9238513532	0.4321194294	-0.9248219308
B	1.9800165875	-0.0948846232	-1.5788167578		B	2.0642531831	0.0068781651	-1.5167812696
B	-2.7959787945	1.3214507701	0.7248179085		B	-2.7967632653	1.2567208121	0.7084612138
B	0.2630014223	-0.2862614401	-0.6968259351		B	0.3165079935	-0.1617070557	-0.7287465789
B	-0.2045465696	0.1345778421	0.9351958867		B	0.0334651317	0.4209625356	0.9565750696
B	3.2331925043	-0.9528015358	-0.503097091		B	3.2172771901	-1.0143939184	-0.4835093873
B	3.0456721688	0.1916909654	1.0491047344		B	3.1453538131	-0.094338308	1.1282028918
B	-1.8649661311	0.2295637027	1.7672701711		B	-1.719164165	0.3779334793	1.8131608618
B	-3.2976500779	-0.3538071393	0.8120218176		B	-3.0579875549	-0.4589395186	0.9369435974
B	1.5759370893	-1.526637585	-0.7218171333		B	1.514177854	-1.4592227948	-0.7999580771
B	1.4355888083	-0.2308309045	1.8736648661		B	1.4132852404	-0.3116782177	1.8749059966
B	0.6263748525	-1.3371034663	0.8072795588		B	0.488701313	-1.2613085042	0.6822639545
B	2.3636612673	-1.4455336055	0.9456065169		B	2.2266314593	-1.5370921642	0.8841846929
S	3.2182257623	0.9962731459	-0.6956844584		S	3.2636818057	0.9550448994	-0.4447359107
S	-3.1953669404	-1.4125627614	-0.861415384		S	-2.8734243045	-1.556697366	-0.7035624264
H	-3.8469741336	0.9130856417	-1.6143140132		H	-3.8732092995	0.6846638048	-1.5878624546
H	-4.3195260594	-0.5907539597	1.3612677751		H	-4.0091588011	-0.8163193443	1.5466233817
H	-3.5921808167	2.114178383	1.1057488515		H	-3.6420274778	2.0021893035	1.0808159424
H	-1.9689653374	0.2018051799	2.9493139095		H	-1.8117019642	0.439248203	2.9941025646
H	-0.7794060568	2.4872901838	1.8472064684		H	-0.8733195606	2.6955288133	1.6405712558
H	-2.00471983	2.8306988815	-1.1629398967		H	-2.1824518976	2.7537001288	-1.2536964175
H	-0.8427379028	0.8112662467	-2.8436299494		H	-0.8996704476	0.7491775761	-2.8828624014
H	0.7874968483	2.2317037993	-0.5013741477		H	0.6814541339	2.4052737718	-0.7529304668
H	1.4311181848	-0.3051463676	3.0565612761		H	1.3024010873	-0.4601610886	3.0452805775
H	0.0080759511	-2.2731169718	1.2031014793		H	-0.247956507	-2.1498181392	0.9511165835
H	1.3513750369	-2.5196279521	-1.3356492615		H	1.2607766116	-2.3862714847	-1.497541583
H	1.9499925195	-0.0020629615	-2.7583793156		H	2.1176571764	0.2294997414	-2.677632958
H	4.1978194849	-1.5339169624	-0.8631437277		H	4.153490003	-1.6035373081	-0.8995324676
H	2.7895325083	-2.4175539248	1.473734897		H	2.5264994699	-2.5312898506	1.4588742628
H	3.9583938652	0.4557824868	1.7558250002		H	4.0688558932	0.0355128322	1.858346148
H	1.8647643529	0.8951904884	1.48418219		H	1.1724012621	0.9125874289	1.5713093809
H	-2.1885689533	-0.9529896882	1.3695988364		H	-1.9026839996	-0.8736821064	1.5238492991
H	-0.6848659096	-0.6549445166	-1.5033867213		H	-0.6861223783	-0.6185718608	-1.4542780869
H	-1.9688833788	-1.9519419571	-0.7064379103		H	-4.2016268077	-1.7572238895	-0.8194894298

References

1. Bould, J.; Ehn, M.; Tok, O.; Bavol, D.; Kučeráková, M.; Clegg, W.; Litecká, M.; Lang, K.; Kirakci, K.; Londesborough, M. G. S., Expanding Luminescence Horizons in Macropolyhedral Heteroboranes. In *Angew. Chem., Int. Ed. Engl.*, anie202401872-sup-0001-misc_information.pdf, 2024.
